# Supplementary material for: Shifting molecular localization by plasmonic coupling in a single-molecule mirage
Source: Nat Commun. 2017 Jan 11;8:13966. doi: 10.1038/ncomms13966 (PMC5512867; doi:10.1038/ncomms13966)
Supplement: Supplementary Information — Supplementary Figures, Supplementary Tables, Supplementary Note and Supplementary References. [file ncomms13966-s1.pdf]

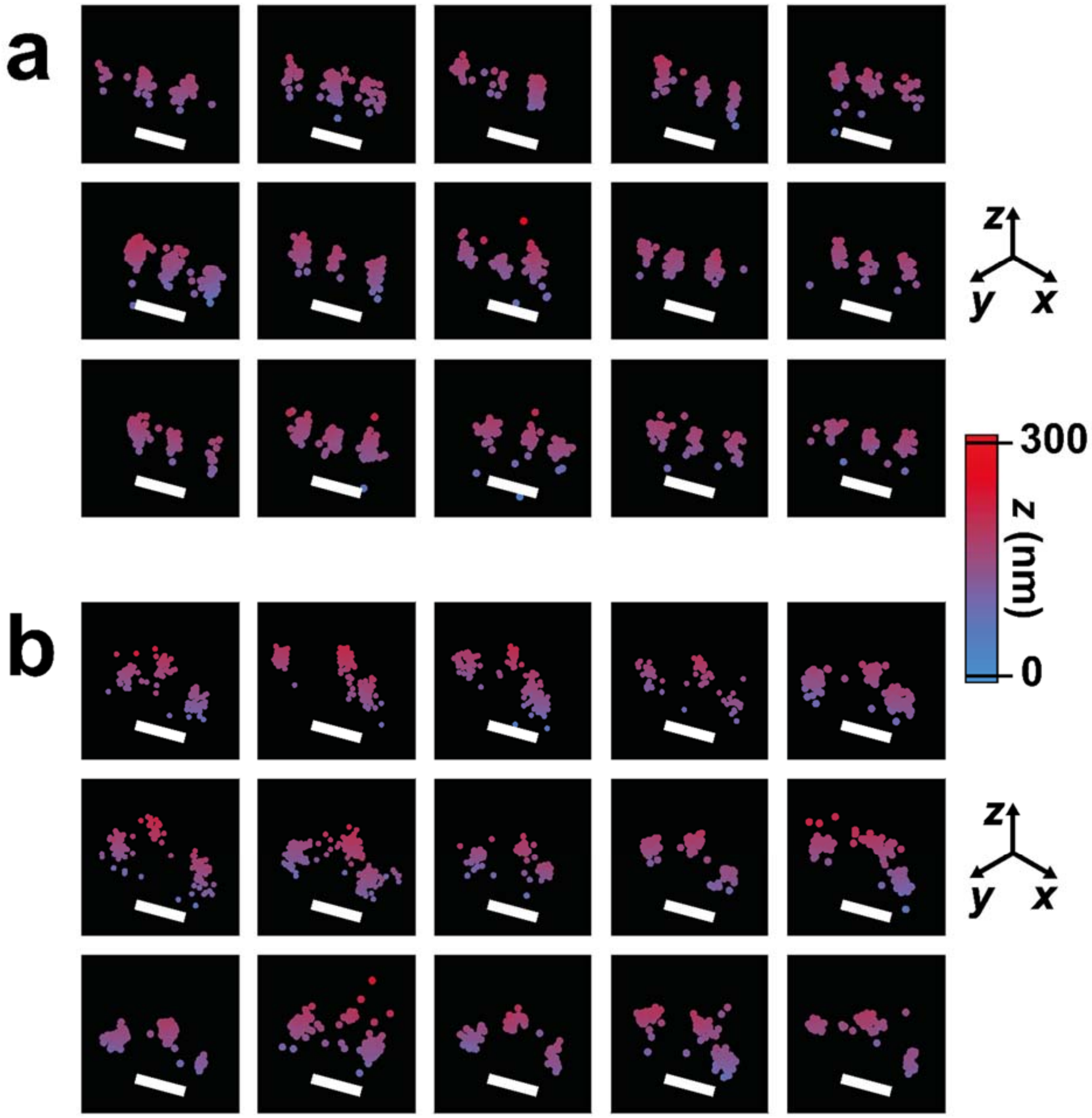

Supplementary Figure 1 | 3D images of the 12 helix bundle. Additional 3D-DNA-PAINT-Images (points accumulation for imaging in nanoscale topography) of 12 helix bundle DNA origamis without (a) and with an 80 nm gold-nanoparticle above their middle (b). While the images in (a) just show straight lines in space the images in (b) show a triangular shape due to plasmonic coupling. Scale bars 100 nm.

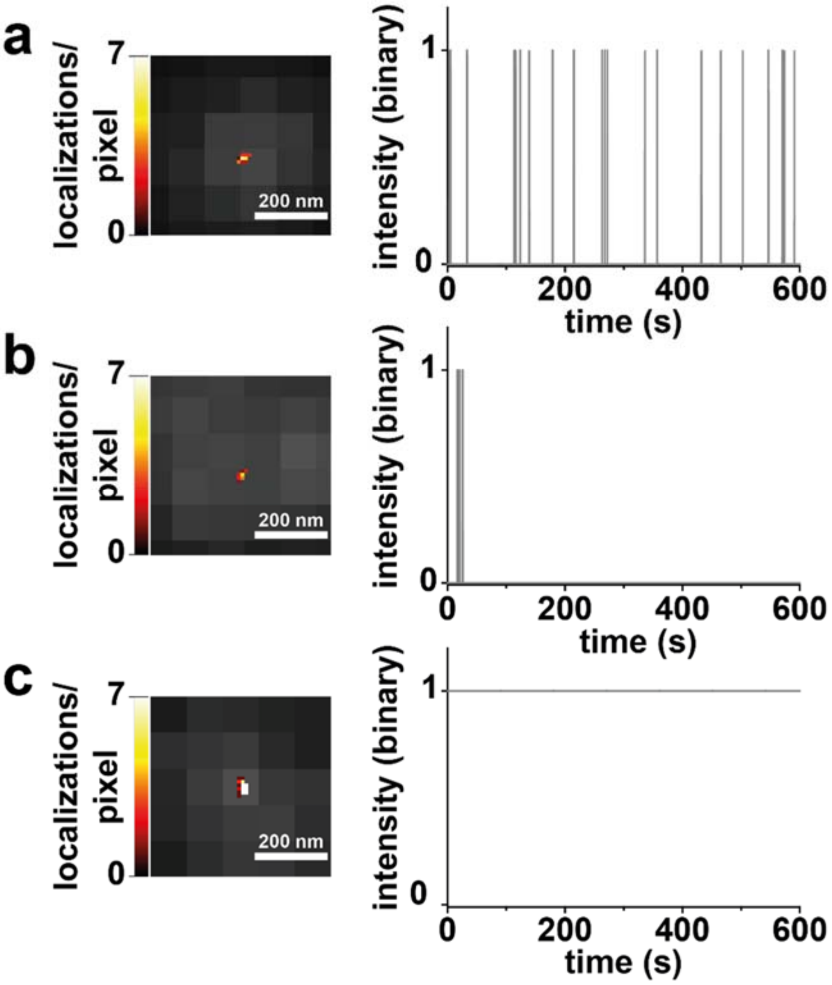

Supplementary Figure 2 | Analysis of transients. In order to assure that all localization events considered corresponded to DNA-PAINT (points accumulation for imaging in nanoscale topography), we analyzed the time traces of each structure. DNA-PAINT events are easily identified because they show repetitive bursts of fluorescence during the whole measurement time. Fluorophores bound unspecifically provide one or a few bursts until photobleaching. Such events were discarded for the analysis as well as signals from aggregates of gold nanoparticles (AuNP) that are constant in time and constitute a minor fraction of the colloidal suspension. Exemplary fluorescence transients of DNA-PAINT (a), an unspecifically bound fluorophore (b), and scattering of AuNP aggregates (c) are shown.

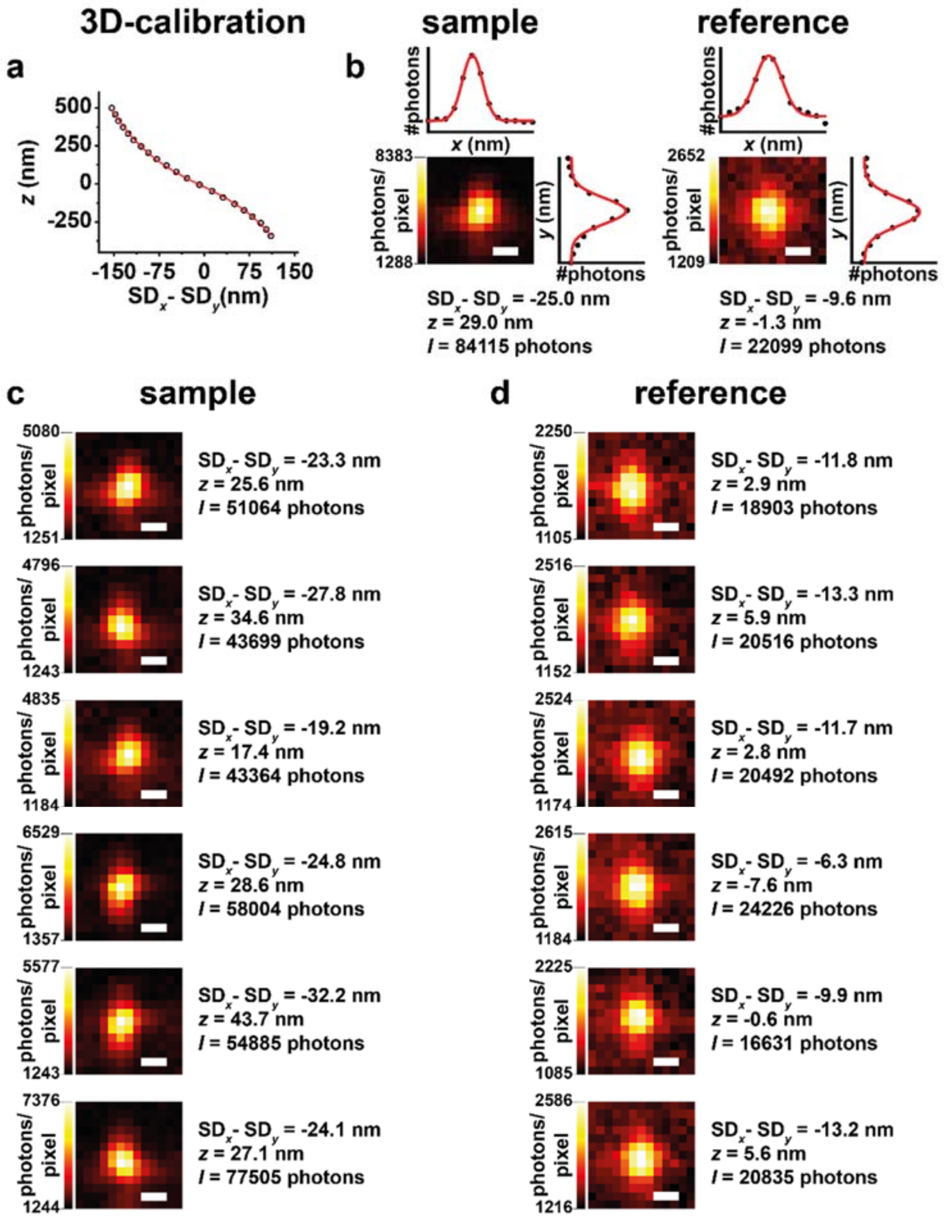

Supplementary Figure 3 | 3D superresolution calibration. a) The calibration for calculating the axial coordinates by using a cylindrical lens to blurr the standard deviation (SD) from the point spread function (PSF) was carried out with TetraSpeck-Beads (100 nm, Invitrogen, T7279) according to a protocol described by Schmied et al[1]. b) An analysis of two PSFs for a reference structures as well as for a sample carrying an 80 nm gold nanoparticle. The intensity *I* is the background-corrected integral over the PSF. c, d) Further examples of PSFs indicate that the NPs do not interfere with the 3D determination of the emission centre as was reported for more complex nanostructures such as nanowires[2]. Interestingly, as also indicated in the graph, we found enhanced photon emission for dyes near the 80 nm AuNPs in accordance with recent reports.[3] Scale bars 300 nm.

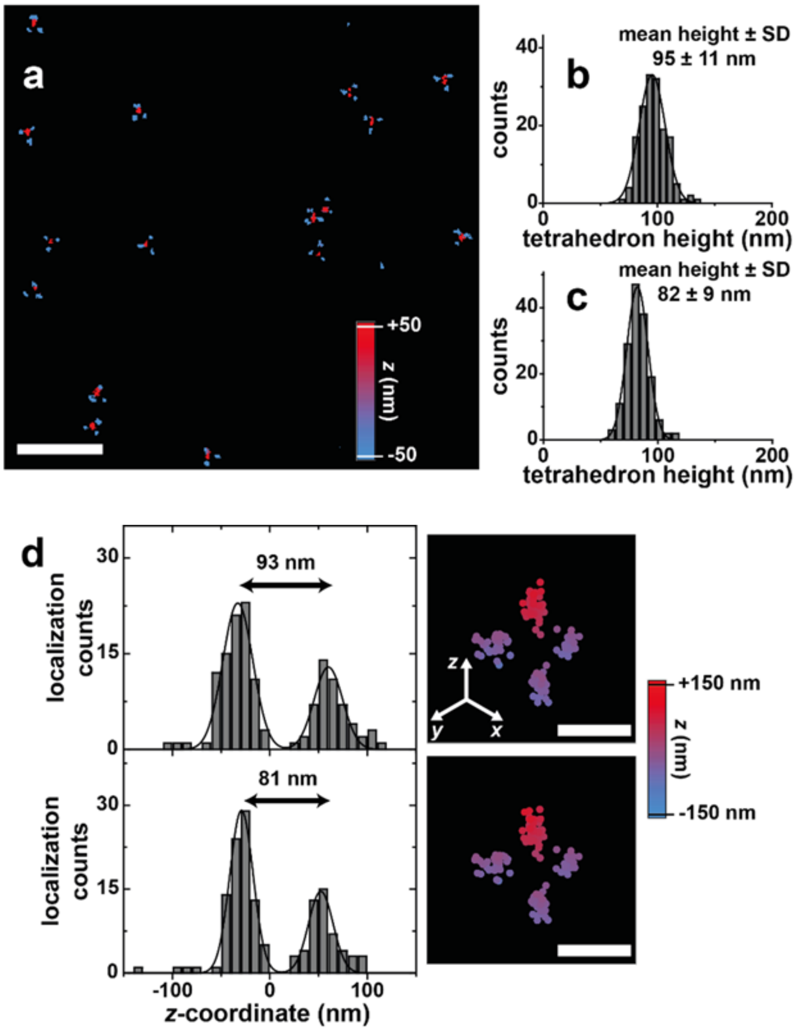

Supplementary Figure 4 | Experimental determination of the 3D-correction-factor using tetrahedral DNA-origamis. Due to the refractive-index-mismatch between the calibration sample and the experimental samples, a linear correction factor was applied for the axial localizations[1,4,5]. Determination of the correction factor requires a second calibration step with single molecules at known *z*-position. For this purpose we performed DNA-PAINT (points accumulation for imaging in nanoscale topography) imaging of tetrahedral DNA origami structures of well-defined height (82 nm) introduced by Iinuma et al[6] and determined the correction factor by calculating the quotient of expected and measured height. For our experiment, this correction factor is 0.86. a) Super-resolution 3D-DNA-PAINT-image of the tetrahedrons. Scale bar 500 nm. b) Statistics over the uncorrected tetrahedron heights (SD = standard deviation). c) Statistics over the tetrahedron heights after correcting the *z*-coordinates with a factor of f = 0.86. d) 3D-scatter-plots of an uncorrected (top) and a corrected tetrahedron with corresponding *z*-localization histogram. Scale bars for c and d are 100 nm.

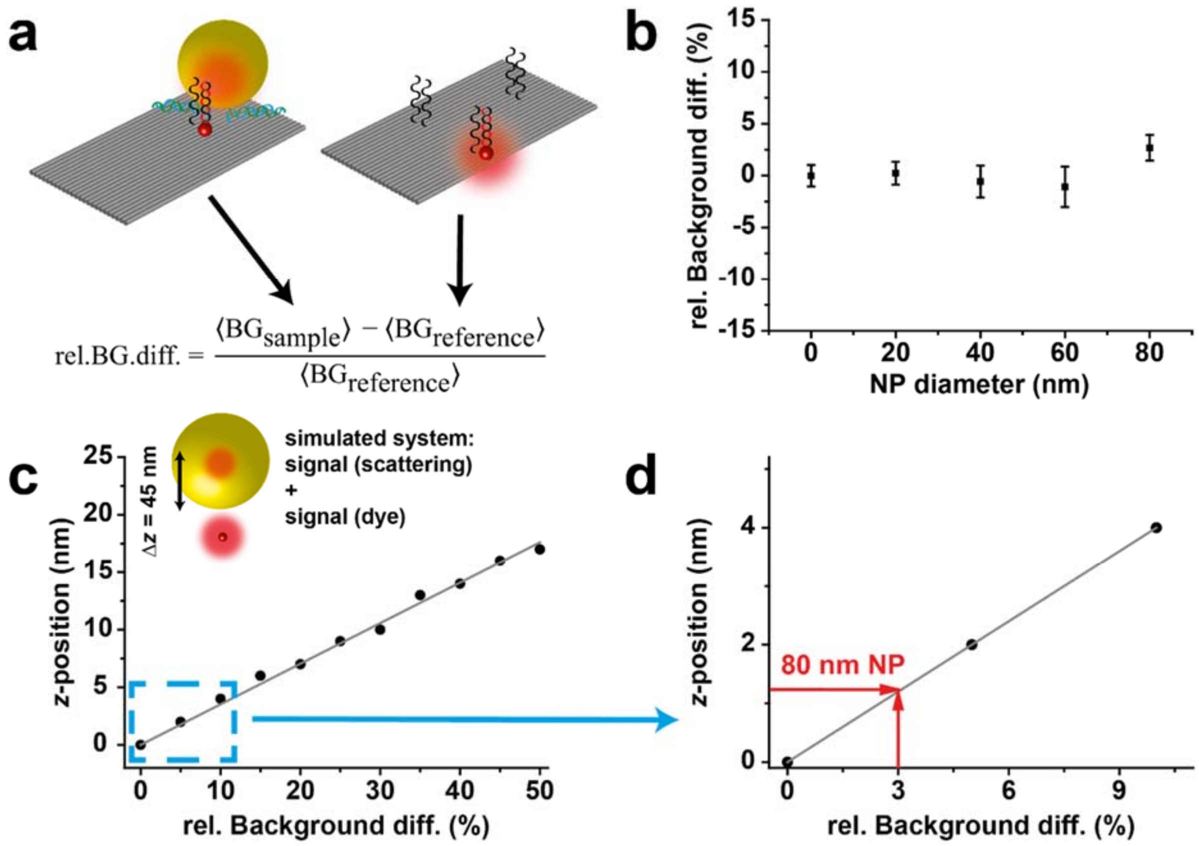

Supplementary Figure 5 | Contribution of constant scattering signals to *z*-position determination. a) Formula to calculate the relative background (BG) difference between DNA origamis imaged without gold nanoparticles (AuNPs) attached compared to those with AuNPs. b) Single AuNPs of 20 nm, 40 nm, and 60 nm produced negligible scattering in the spectral region of fluorescence detection. The average scattering of the 80 nm AuNPs is only 3% larger than the average background signal of Atto655 (error bars are standard deviations). c) The potential contribution of that continuous scattering signal to a mislocalization was accounted for via Monte Carlo simulations. We simulated photon detection counts from the fluorophore and a second emitter placed 45 nm above (5 nm DNA spacer and 40 nm of the AuNP radius) with different relative intensities. Photon counts were distributed over 2D Gaussians, binned at 100 nm pixels. The simulated signals were analyzed for localization using the same algorithms used for the experimental data. As expected, the detected axial position increases with the scattering intensity (here in terms of the relative BG-intensity as in the experiment) in an approximately linear way. d) an inset of the relevant area (blue box in c). The background signal of the 80 nm AuNPs can only lead to a signal shift of ~1 nm which is insignificant for our experimental determinations.

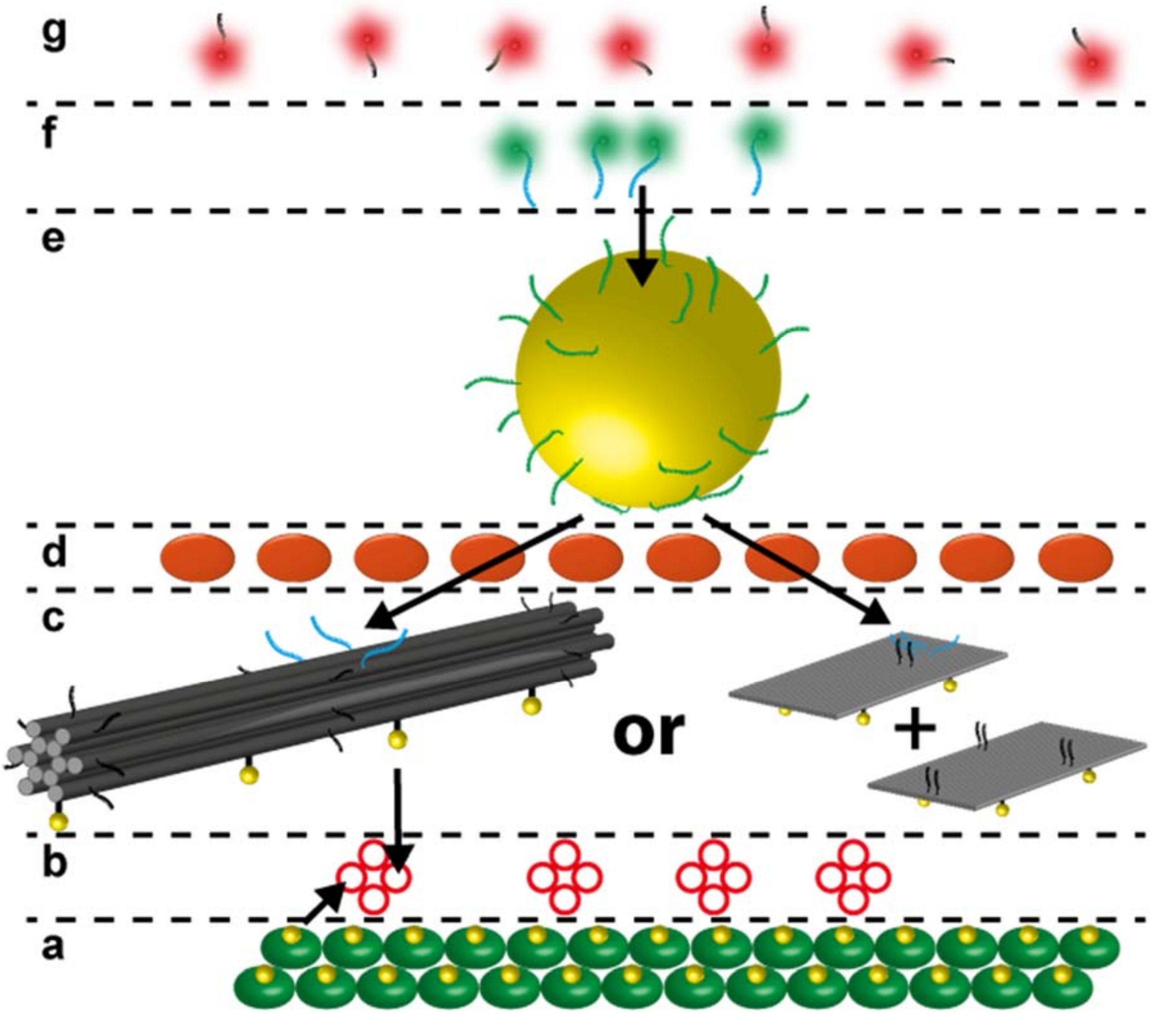

Supplementary Figure 6 | Sample preparation. All samples were prepared by a multi-step self-assembly procedure as follows: a) First a monolayer of biotin-modified bovine serum albumin (BSA) was deposited on a glass substrate. b) Next a NeutrAvidin layer was deposited. c) The DNA origami assembled onto the NeutrAvidin layer from a specific side through biotin anchors included in the DNA origami. The DNA origami structures included docking sites for DNA-modified gold nanoparticles (AuNPs) as well as sites for DNA-PAINT (points accumulation for imaging in nanoscale topography). d) The remaining NeutrAvidin surface was passivated by incubation with Superblock ®. e) AuNPs surface modified with single stranded DNA were attached to the DNA origami. f) The AuNPs smaller than 60 nm were subsequently labeled with Cy3B fluorophores. g) Finally the buffer containing the Atto655-labeled oligonucleotides for DNA-PAINT imaging was introduced.

Supplementary Table 1 │ Range of gold nanoparticle (AuNP)-fluorophore distances and AuNP size distributions. The separation distance between the AuNPs and the fluorophores was estimated according to the DNA origami design, considering a diameter of 3 nm for the DNA double-helices and a separation of 0.34 nm between two adjacent base pairs. An overall position uncertainty of 3 nm was taken into account according to previous experiments[7]. Since the DNA-PAINT (points accumulation for imaging in nanoscale topography) markers and the AuNPs capturing strands are placed at a fixed position on the origami structure, the resulting NP-fluorophore distance will depend on the NP size, as summarized in this table. We also took into account the size distribution of the AuNPs, which affects not only the AuNP-fluorophore separation distance but also the electromagnetic coupling[8].

NP-diameter (nm)	mean NP-fluorophore distance (nm)
20	9.1
40	7.7
60	7.0
80	6.6

Supplementary Table 2 | Staple strands of the 12 helix bundle (12 HB)

Oligo	Sequence (from 5‘ to 3‘)
1	AAAGGGCGCTGGCAAGTATTGGC
2	TCAGAGGTGTGTCGGCCAGAATGAGTGCACTCTGTGGT
3	GGCATAAGCGTCTTCGAGGAAACGCA
4	TACATAAATTCTGGGCACTAACAACT
5	CAATCCAAAATACTGAACAGTAG
6	CATAGTTAATTTGTAAATGTCGC
7	GAACAAGAGTCCACCAATTTTTTAGTTGTCGTAGG
8	TTGAAGCCCTTTTTAAGAAAAGT
9	AAGCACAGAGCCTAATTATTGTTAGCGATTAAGACTCCTT
10	GCGCCTGAATGCCAACGGCCCAGCCTCCCGCGTGCCTGTTCTTCTTTTT
11	TTGACGGGGAAAGCTTCACCAGAAATGGCATCACT
12	CATTCAACCCAAAATGTAGAACCCTCATGAATTAGTACAACC
13	GATGTTTTTCTTTTCACCA
14	TCCCATCCTAATGAGAATAACAT
15	ATCAGCGGGGTCAGCTTTCAGAG
16	TTCGCTATTCGCAAGACAAAGTTAATTTCATCTTC
17	TTGAGAATATCTTTCCTTATCACTCATCGAGAACA
18	GGGCGTGAAATATTAGCGCCATTCGC
19	GGCGCCCCGCCGAATCCTGAGAAGTGAGGCCGATTAAAGG
20	TTTTTTGTTTAATAAAGTAATTC
21	AAATCAGCCAGTAATAACACTATTTTTGAAGCCTTAAATC
22	GGTCACGCCAGCACAGGAGTTAG
23	TGAACAGCTTGATACCGATAGTT
24	AAAATTCCATTCAGGCTTTTGCAAAA
25	AGCACTAAATCGGATCGTATTTAGACTTATATCTG
26	AGACGGGAGAATTGACGGAAATT
27	TAAGCCAGAGAGCCAGAAGGAAACTCGATAGCCGAACAAA
28	CGCCTGACGGTAGAAAGATTCTAATGCAGATACAT
29	CAGTCTTGATTTTAAGAACTCAACGTTGCGTAT
30	CATAGAATTTGCGGTTTGAAAGAGGA
31	GCGCAGCGACCAGCGATTATATATCATCGCCTGAT
32	TTTTTAAAAACGCTCATGGAAATA
33	AATCAGTTAAAACGTGGGAGAAA

34	GGTGCCGTCGAGAGGGTTGATAT
35	GTCAGAATCAGGCAGGATTCGCG
36	TTTTTTATAACGTGCTTTCCTCTTTATAACAGTACTAT
37	AGACAACCTGAACAGTATTCGAC
38	CCGAACGGTGTACAGACCAGGCG
39	ATTCAAGGGGAAGGTAAATGTGGCAAATAAATC
40	GTCACCAGTACAAGGTTGAGGCA
41	TAAATCGGTTGGTGCACATCAAAAATAA
42	AGACGGCGAACGTGGCGAG
43	CCCTTCATATAAAAGAACGTAGAGCCTTAAAGGTGAATTA
44	AACTTTAATCATGGGTAGCAACG
45	ACCATCACCCAAATAAACAGTTCATTTGATTCGCC
46	TTTGCAACCAGCTTACGGCGGTGGTGAGGTTTCAGTTGAGGATCCTTTTT
47	TGCAACACTATCATAACCCTCGT
48	AACGAACCTCCCGACTTGCGGGA
49	TGCCTAATGAGTGAGAAAAGCTCATATGTAGCTGA
50	GGTTTGCGCATTTTAACGCGAGGCGT
51	AAAAGAATAGCCCGATACATACGCAGTAAGCTATC
52	TTTCACGAGAATGACCATTTTCATTTGGTCAATAACCTGT
53	TCGGTCATACCGGGGGTTTCTGC
54	CCTCCGAAATCGGCAAAAT
55	TTCCATTGACCCAAAGAGGCTTTGAGGA
56	ACGCGTCGGCTGTAAGACGACGACAATA
57	GTCCGTCCTGCAAGATCGTCGGATTCTCTTCGCATTGGACGA
58	TTTTTTGGTAATGGGTAACCATCCCACTTTTT
59	GGAGCAGCCACCACCCTTCGCATAACGACAATGACAACAA
60	AAAAGTGTCAGCAACAATTGCAGGCGCT
61	GTCAGTCGTTTAACGAGATGGCAATTCA
62	AATGCTGTAGCTGAGAAAGGCCG
63	CTATATTAAAGAACGTGGA
64	CGGTAGTACTCAATCCGCTGCTGGTCATGGTC
65	CTTGAAAACACCCTAACGGCATA
66	AAGTAAGAGCCGCCAGTACCAGGCGG
67	AAAAGATAGGGTTGAGTGT
68	TTCGCCATAAACTCTGGAGGTGTCCAGC
69	AGGGCGAAAAACCGATTTAACGTAGGGCAAATACC
70	GAGCTTAAGAGGTCCCAATTCTGCAATTCCATATAACAGT
71	GCAGCACTTTGCTCTGAGCCGGGTCACTGTTGCCCTGCGGCTTTTT
72	TACCTGGTTTGCCCCAGCA
73	CCCACATGTGAGTGAATAACTGATGCTTTTAACCTCCGGC
74	ACAGCTGATTGCCCGTCGCTGCGCCCACACGTTGA
75	ATTAAAATAAGTGCGACGATTGGCCTTG
76	AAAACGAAAGAGGCTCATTATAC
77	TGTCCAAGTACCAGAAACCCCAG
78	TTACCAATAAGGCTTGCAGTGCGGAAGTTTAGACTGGATA
79	TTAGTGTGAATCCCTCTAATAAAACGAAAGAACGATGAATTA
80	ATCAGAGCCTTTAACGGGGTCTTAATGCCCCCTGC
81	TTACCTCTTAGCAAATTTCAACCGATTG
82	TTTTTAGGAGCGGGCGCTAGGAAGGGAAGAAAGCGAATTTTT
83	TGCCATACATAAAGATTAACTGAACACCAACAGCCGGAATAG
84	TTTTTCCGGTGCAGCACCGATCCCTTACACTTGCC
85	AAAACGGAATACCCAAAAGAACT
86	GCTAAATCGGTTTGACTATTATA
87	CAGCTTTGAATACCAAGTTACAA
88	GGTTGCTTTGACGAGCACGTTTTT
89	CATGCCAGTGAGCGCTAATATCCAATAATAAGAGC
90	TATGCATTACAGAGGATGGTTTAATTTC
91	ACTGCCCGCTTTCCTGAAAAGCTATATTTTAAATA
92	TGATTTAGAAAACTCAAGAGTCAATAGT

93	TGGGCGCCAGGGTGATTCATTAGAGTAACCTGCTC
94	GTCCACGCGCCACCTCACCGTTGAAACA
95	TTTTTATCCAGCGCAGTGTCACTGC
96	GATGAATAAATCCTGTAGGTGAGGCGGTAGCGTAAGTCCTCA
97	TGCAACTCAAAAGGCCGTACCAAAAACA
98	GTTTGATGGTGGTTCAGAACCCCGCCTCACAGAAT
99	TCACCGTCACCGGCGCAGTCTCT
100	AGACGTCGTCACCCTCAGATCTTGACGCTGGCTGACCTTC
101	TTTAGCAAACGCCACAATATAACTATATTCCCTTATAAATGG
102	AGCGTATCATTCCACAGACCCGCCACAGTTGCAGCAAGCG
103	GTATGTGAAATTGTTATCC
104	CCGAACTTTAATAAAAGCAAAGCGGATT
105	GTGAGTTAAAGGCCGCTGACACTCATGAAGGCACCAACCT
106	AAATAGGTAATTTACAAATAAGAAACGA
107	TGTTCCAACGCTAACGAACAAGTCAGCAGGGAAGCGCATT
108	GTGCCTGCTTTAAACAGGGAGAGAGTTTCAAAGCGAACCA
109	GCGCCCGCACCCTCTCGAGGTGAATT
110	TAAAGAGGCAAAATATTTTATAA
111	GTTTACCGCGCCCAATAGCAAGC
112	TACCGGGATAGCAATGAATATAT
113	AAATTGTGTCGAGAATACCACAT
114	AAATGCGTTATACAAATTCTTAC
115	CAGATATAGGCTTGAACAGACGTTAGTAAAGCCCAAAAATTT
116	TAAGATCTGTAAATCGTTGTTAATTGTAAAGCCAACGCTC
117	CATTCTATCAGGGCGATGG
118	ACAGTTTTTCAGATTTCAATTACCGTCGCAGAGGCGAATT
119	TTTAGAACGCGAATTACTAGAAAACTATAAACACCGGAAT
120	TGACCTAAATTTTTAAACCAAGT
121	CTCCAATTTAGGCAGAGACAATCAATCAAGAAAAATAATA
122	CATCGGGAGAAATTCAAATATAT
123	ATCATTTACATAAAAGTATCAAAATTATAAGAAACTTCAATA
124	GCTACGACAGCAACTAAAAACCG
125	TTAGGTTGGGTTATAGATAAGTC
126	TATTGCCTTTAGCGTCAGACTGT
127	TTTTTCCGGGTACCGAGCTCGAATTCGTAATCTGGTCA
128	CTAAAGACTTTTAGGAACCCATG
129	GTGGAACGACGGGCTCTCAACTT
130	GAGACAAAGATTATCAGGTCATTGACGAGAGATCTACAAA
131	AGGGACAAAATCTTCCAGCGCCAAAGAC
132	AAAATTTTTTAAAATGAGCAAAAGAA
133	TCAGGTGAAATTTCTACGGAAACAATCG
134	ATAATGAATCCTGAGATTACGAGCATGTGACAAAAACTTATT
135	GAGGTAACGTTATTAATTTTAAAACAAATAATGGAAGGGT
136	ACCGCATTCCAACGGTATTCTAAGCGAGATATAGAAGGCT
137	CAGCATCAACCGCACGGCGGGCCGTT
138	GCTCAAGTTGGGTAACGGGCGGAAAAATTTGTGAGAGATA
139	GGAATCGGAACATTGCACGTTAA
140	ATAAGAAGCCACCCAAACTTGAGCCATTATCAATACATCAGT
141	GGCGACACCACCCTCAGGTTGTACTGTACCGTTCCAGTAA
142	AAGACGCTGAGACCAGAAGGAGC
143	AGCAGTCGGGAAACCTGTC
144	AACAACATGTTCATCCTTGAAAA
145	CATGTCAGAGATTTGATGTGAATTACCT
146	TATGTGATAAATAAGGCGTTAAA
147	TTAATGAATCGGCCATTCATTCCAATACGCATAGT
148	ATTCTTTTCATAATCAAAATCAC
149	AATCGTTGAGTAACATTGGAATTACCTAATTACATTTAAC
150	ATTTTGCCAGAGGGGGTAATAGT
151	AGCGCCACCACGGAATACGCCTCAGACCAGAGCCACCACC

152	AAAAAAGGCAGCCTTTACAATCTTACCAGTTTG
153	TAATCGTAGCATTACCTGAGAGTCTG
154	AATAGCTGTCACACGCAACGGTACGCCAGCGCTTAATGTAGTA
155	GCAGCACCGTAAGTGCCCGTATA
156	ATGAATCCCAGTCACGATCGAACGTGCCGGCCAGAGCACA
157	CAAGTGCTGAGTAAGAAAATAAATCCTC
158	TCAACATCAGTTAAATAGCGAGAGTGAGACGACGATAAAA
159	AATAACGCGCGGGGAGAGG
160	AAGAGATTCATTTTGTTTAAGAGGAAGC
161	CAAATGGTTCAGAAGAACGAGTAGAT
162	AAAAGGGCGACAATTATTTATCC
163	ATAGCTGTTTCCTGGAACGTCCATAACGCCGTAAA
164	TGTAGGGGATTTAGTAACACTGAGTTTC
165	AAAAATCTACGTGCGTTTTAATT
166	GGCTAAAGTACGGTGTCTGGAAG
167	CCTACATACGTAGCGGCCAGCCATTGCAACAGGTTTTT
168	CTATTTCGGAACGAGTGAGAATA
169	AGAGTTTATACCAGTAGCACCTGAAACCATCGATA
170	ACTACCTTTAAACGGGTAACAGGGAGACGGGCA
171	AATCCAAAAAAAAGGCTCCAAAA
172	GAGAGCCTCAGAACCGCATTTTCTGTAACGATCTAAAGTT
173	AAATCCCCGAAACAATTCATGAGGAAGT
174	TACCTAATATCAAAATCATTCAATATTACGTGA
175	GTATACAGGTAATGTGTAGGTAGTCAAATCACCAT
176	AACGTTGTAGAAACAGCGGATAGTTGGGCGGTTGT
177	GTTTATGTCACATGGGAATCCAC
178	GTGTATTAAGAGGCTGAGACTCC
179	GAAGTCAACCCAAATGGCAAAAGAATACTCGGAACAGAATCC
180	CGGTTAACAAAGCTGCTGTAACAACAAGGACGTTGGGAAG
181	ATATTCACAAACAAATTCATATG
182	TTCATTTTCTGCTAAACAACTGAACAACTAAAGGA
183	TCGTTCACCGCCTGGCCCT
184	CGGAAGCACGCAAACTTATTAGCGTT
185	GAGCAAGGTGGCATTTACTCCAACAGGTTCTTTACGTCAACA
186	ATTGCGAATAATGTACAACGGAG
187	CTTTTTTTCGTCTCGTCGCTGGC
188	GACCGTCGAACGGGGAAGCTAATGCAGA
189	GCGTCATACATGCCCTCATAGTT
190	GACCGGAAGCAATTGCGGGAGAA
191	TCAAGCAGAACCACCACTCACTCAGGTAGCCCGGAATAGG
192	AGCCTCCCCAGGGTCCGGCAAACGCG
193	GAAAGTTCAACAATCAGCTTGCTTAGCTTTAATTGTATCG
194	TAGAACCTACCAGTCTGAGAGAC
195	GGGTTACCTGCAGCCAGCGGTGTTTTT
196	GAATTATCCAATAACGATAGCTTAGATT
197	TTGTCGTCTTTCTACGTAATGCC
198	ACTACTTAGCCGGAACGAGGCGC
199	TTTTTGTCCATCACGCAAATTCCGAGTAAAAGAGTCTTTTTT
200	TTTTTCGGGAGCTAAACAGGTTGTTAGAATCAGAGTTTTT
201	AATCATAATAACCCGGCGTCAAAAATGA
202	TGTAAATCATGCTCCTTTTGATAATTGCTGAATAT
203	TTCACCTAGCGTGGCGGGTGAAGGGATACCAGTGCATAAAAA
204	ATTTGCCAAGCGGAACTGACCAACGAGTCAATCATAAGGG
205	AGCAAGCCGTTTAAGAATTGAGT
206	GCCCGCACAGGCGGCCTTTAGTG
207	CAGTAAGAACCTTGAGCCTGTTTAGT
208	ACCAAATTACCAGGTCATAGCCCCGAGTTTTCATCGGCAT
209	TCTTATACTCAGAAAGGCTTTTGATGATATTGACACGCTATT
210	GCCTTATACCCTGTAATACCAATTCTTGCGCTC

211	TTTTTGCGTCCGTGCCTGCATCAGACGTTTTT
212	TTATGGCCTGAGCACCTCAGAGCATAAA
213	CGAGCACAGACTTCAAATACCTCAAAAGCTGCA
214	AACAGAGTGCCTGGGGTTTTGCTCACAGAAGGATTAGGAT
215	CCAGCCAAACTTCTGATTGCCGTTTTGGGTAAAGTTAAAC
216	TGAAATTGTTTCAGGGAACTACAACGCC
217	GCATCAAAAAGAAGTAAATTGGG
218	GAATTGTAGCCAGAATGGATCAGAGCAAATCCT
219	GCTTGACCATTAGATACATTTCG
220	CTGAAAACCTGTTTATCAAACATGTAACGTCAA
221	GACTTTCTCCGTGGCGCGGTTG
222	ACACAACATACGAGGGATGTGGCTATTAATCGGCC
223	TTTTTAACAATATTACCGTCGCTGGTAATATCCAGTTTTT
224	TGCCTGAACAGCAAATGAATGCGCGAACT
225	CAAATATCAAACCAGATGAATAT
226	TAAGTAGAAGAACTCAAACTATCG
227	ATTTGGCAAATCAACAGTTGAAA
228	GTTGAAACAAACATCAAGAAAAC
229	CAATATGATATTGATGGGCGCAT
230	GTTTGAGGGGACCTCATTTGCCG
231	GTATTAGAGCCGTCAATAGATAA
232	GCTAATGCCGGAGAGGGTAGCTA
233	TACTTCTTTGATAAAAATCTAAA
234	GAAAGATCGCACTCCAGCCAGCT
235	TCAGGCTGCGCAACTGTTGGGAA
236	ATACCCTTCGTGCCACGCTGAACCTTGCTGAACCT
237	CATAATATTCCGTAATGGGATCCGTGCATCTGCCA
238	TTCTGGAATAATCCTGATTTTGCCCGGCCGTAA
239	TTAACAAGAGAATCGATGAACGG
240	GGGCCGGAAGCATAAAGTG
241	TTTTTATCCAATAAATCTCTACCCCGGTAAAACTAGCATG
242	CCGGAAGACGTACAGCGCCGCGATTACAATTCC
243	TTCGCGGATTGATTGCTCATTTTTTAAC
244	TAAAGGATTGTATAAGCGCACAAACGACATTAAATGTGAG
245	GATAAAAATTTTTAGCCAGCTTT
246	GATAGTGCAACATGATATTTTTGAATGG
247	GGATAACCTCACAATTTTTGTTA
248	TCAATAATAAAGTGTATCATCATATTCC
249	CAATAGGAACGCAAATTAAGCAA
250	CCGATAATAAAAGGGACTTAACACCGCGAACCACCAGCAG
251	CATCAGCGTCTGGCCTTCCACAGGAACCTGGGG
252	GGAATAACAGAGATAGACATACAAACTTGAGGATTTAGAA
253	GCGAAAGACGCAAAGCCGCCACGGGAAC
254	AACACCCTAAAGGGAGCCC
255	GCATCGAGCCAGATATCTTTAGGACCTGAGGAAGGTTATC
256	CGTAAAGGTCACGAAACCAGGCAATAGCACCGCTTCTGGT
257	CGAGTAACAACCGTTTACCAGTC
258	GCCTTACGCTGCGCGTAAAATTATTTTTTGACGCTCAATC
259	CCGAACCCCCTAAAACATCGACCAGTTTAGAGC
260	TGCGTACTAATAGTAGTTGAAATGCATATTTCAACGCAAG
261	GATTTTAGACAGGCATTAAAAATA
262	TTCCGAATTGTAAACGTGTCGCCAGCATCGGTGCGGGCCT
263	ACATCATTTAAATTGCGTAGAAACAGTACCTTTTA
264	AAGATAAAACAGTTGGATTATAC
265	TGATTATCAGATATACGTGGCAC
266	TGGCAAGTTTTTTGGGGTC
267	TCAGCTAACTCACATTAAT
268	CTATTAGTCTTTCGCCGCTACAG
269	AACGCCAAAAGGCGGATGGCTTA

270	AAGAAACAATGACCGGAAACGTC
271	GTACATCGACATCGTTAACGGCA
272	ATACCACCATCAGTGAGGCCAAACCGTTGTAGCAA

Supplementary Table 3 | Modifications to the 12 helix bundle (12 HB)

**Modified staple positions on the 12 HB**
DNA PAINT docking strands on 3': 23, 34, 40, 75, 80, 151, 155, 168, 178, 181, 191, 193, 214, 225, 227, 230, 231, 232, 234, 235, 236, 237, 240, 243, 244, 246, 247, 250, 251, 252, 254, 255, 256, 259, 262, 268
Docking-strands for Gold-NP on 3': 43, 54, 72, 99, 102, 141
Biotins on 3': 8, 198, 206, 226, 269, 270, 271, 272
End-staples (left out to suppress aggregation): 10, 32, 36, 46, 58, 71, 82, 84, 88, 95, 127, 167, 195, 199, 200, 211, 223
**Sequences of modified oligonucleotides and DNA-elongations on the 12 HB**
Docking-strand for Gold-NP on 3': AAAAAAAAAAAAAAAAAAAAAAAAA
DNA PAINT docking strand on 3': TTAAATGCCCG
10nt-Sequence of the imager strand: CGGGCATTTA-Atto655

Supplementary Table 4 | Staple strands of the rectangle

Oligo	Sequence (from 5‘ to 3‘)
1	CATAAATCTTTGAATACCAAGTGTTAGAAC
2	GATGTGCTTCAGGAAGATCGCACAATGTGA
3	GCAATTCACATATTCCTGATTATCAAAGTGTA
4	GATTTAGTCAATAAAGCCTCAGAGAACCCTCA
5	TCACCAGTACAAACTACAACGCCTAGTACCAG
6	CCAATAGCTCATCGTAGGAATCATGGCATCAA
7	GCTTTCCGATTACGCCAGCTGGCGGCTGTTTC
8	AAAGGCCGGAGACAGCTAGCTGATAAATTAATTTTTGT
9	AAATTAAGTTGACCATTAGATACTTTTGCG
10	AAGCCTGGTACGAGCCGGAAGCATAGATGATG
11	TCATTCAGATGCGATTTTAAGAACAGGCATAG
12	GCCATCAAGCTCATTTTTTAACCACAAATCCA
13	TATAACTAACAAAGAACGCGAGAACGCCAA
14	TTGCTCCTTTCAAATATCGCGTTTGAGGGGGT
15	GTATAGCAAACAGTTAATGCCCAATCCTCA
16	AAAGTCACAAAATAAACAGCCAGCGTTTTA
17	GGCCTTGAAGAGCCACCACCCTCAGAAACCAT
18	TTAACGTCTAACATAAAAACAGGTAACGGA
19	AGTATAAAGTTCAGCTAATGCAGATGTCTTTC
20	TCAAATATAACCTCCGGCTTAGGTAACAATTT
21	TTTCGGAAGTGCCGTCGAGAGGGTGAGTTTCG
22	GAGGGTAGGATTCAAAAGGGTGAGACATCCAA
23	TATTAAGAAGCGGGGTTTTGCTCGTAGCAT
24	GCCCTTCAGAGTCCACTATTAAAGGGTGCCGT
25	ATGCAGATACATAACGGGAATCGTCATAAATAAAGCAAAG
26	AGCCAGCAATTGAGGAAGGTTATCATCATTTT
27	TAAATGAATTTTCTGTATGGGATTAATTTCTT
28	AAACAGCTTTTTGCGGGATCGTCAACACTAAA
29	CGGATTCTGACGACAGTATCGGCCGCAAGGCGATTAAGTT
30	GCGCAGACAAGAGGCAAAAGAATCCCTCAG
31	AGAGAGAAAAAAATGAAAATAGCAAGCAAACT
32	GACAAAAGGTAAAGTAATCGCCATATTTAACAAAACTTTT
33	ACACTCATCCATGTTACTTAGCCGAAAGCTGC
34	CTACCATAGTTTGAGTAACATTTAAAATAT
35	TATATTTTGTCATTGCCTGAGAGTGGAAGATTGTATAAGC
36	CGGATTGCAGAGCTTAATTGCTGAAACGAGTA

37	TAAATCATATAACCTGTTTAGCTAACCTTTAA
38	GTACCGCAATTCTAAGAACGCGAGTATTATTT
39	TCTTCGCTGCACCGCTTCTGGTGCGGCCTTCC
40	GCAAGGCCTCACCAGTAGCACCATGGGCTTGA
41	ATTACCTTTGAATAAGGCTTGCCCAAATCCGC
42	CTTATCATTCCCGACTTGCGGGAGCCTAATTT
43	TTATACCACCAAATCAACGTAACGAACGAG
44	GTAATAAGTTAGGCAGAGGCATTTATGATATT
45	CAACCGTTTCAAATCACCATCAATTCGAGCCA
46	GATGGTTTGAACGAGTAGTAAATTTACCATTA
47	GCACAGACAATATTTTTGAATGGGGTCAGTA
48	AGCAAGCGTAGGGTTGAGTGTTGTAGGGAGCC
49	TCCACAGACAGCCCTCATAGTTAGCGTAACGA
50	ATTATACTAAGAAACCACCAGAAGTCAACAGT
51	TAAGAGCAAATGTTTAGACTGGATAGGAAGCC
52	ATACATACCGAGGAAACGCAATAAGAAGCGCATTAGACGG
53	CAACTGTTGCGCCATTCGCCATTCAAACATCA
54	GATGGCTTATCAAAAAGATTAAGAGCGTCC
55	TAGGTAAACTATTTTTGAGAGATCAAACGTTA
56	AGGCAAAGGGAAGGGCGATCGGCAATTCCA
57	ATTATCATTCAATATAATCCTGACAATTAC
58	GAAATTATTGCCTTTAGCGTCAGACCGGAACC
59	AATGGTCAACAGGCAAGGCAAAGAGTAATGTG
60	ATACCCAACAGTATGTTAGCAAATTAGAGC
61	ATAAGGGAACCGGATATTCATTACGTCAGGACGTTGGGAA
62	CACCAGAAAGGTTGAGGCAGGTCATGAAAG
63	ATCCCAATGAGAATTAACTGAACAGTTACCAG
64	CATGTAATAGAATATAAAGTACCAAGCCGT
65	CCAACAGGAGCGAACCAGACCGGAGCCTTTAC
66	GCTATCAGAAATGCAATGCCTGAATTAGCA
67	GACCTGCTCTTTGACCCCCAGCGAGGGAGTTA
68	AGGAACCCATGTACCGTAACACTTGATATAA
69	CAGCGAAACTTGCTTTCGAGGTGTTGCTAA
70	ACAACTTTCAACAGTTTCAGCGGATGTATCGG
71	CAGCAAAAGGAAACGTCACCAATGAGCCGC
72	ACCTTTTTATTTTAGTTAATTTCATAGGGCTT
73	CGATAGCATTGAGCCATTTGGGAACGTAGAAA
74	GCCCGAGAGTCCACGCTGGTTTGCAGCTAACT
75	ATTTTAAAATCAAAATTATTTGCACGGATTCG
76	ACCTTGCTTGGTCAGTTGGCAAAGAGCGGA
77	CTGAGCAAAAATTAATTACATTTTGGGTTA
78	CCTGATTGCAATATATGTGAGTGATCAATAGT

79	TCAATATCGAACCTCAAATATCAATTCCGAAA
80	CTTTAGGGCCTGCAACAGTGCCAATACGTG
81	AATAGTAAACACTATCATAACCCTCATTGTGA
82	TCACCGACGCACCGTAATCAGTAGCAGAACCG
83	GCCCGTATCCGGAATAGGTGTATCAGCCCAAT
84	TGTAGCCATTAAAATTCGCATTAAATGCCGGA
85	TCGGCAAATCCTGTTTGATGGTGGACCCTCAA
86	TGACAACTCGCTGAGGCTTGCATTATACCA
87	CCACCCTCTATTCACAAACAAATACCTGCCTA
88	CCCGATTTAGAGCTTGACGGGGAAAAAGAATA
89	AAGTAAGCAGACACCACGGAATAATATTGACG
90	CACATTAAAATTGTTATCCGCTCATGCGGGCC
91	TTAAAGCCAGAGCCGCCACCCTCGACAGAA
92	ATATTCGGAACCATCGCCCACGCAGAGAAGGA
93	TTCTACTACGCGAGCTGAAAAGGTTACCGCGC
94	AACGTGGCGAGAAAGGAAGGGAAACCAGTAA
95	GAATTTATTTAATGGTTTGAAATATTCTTACC
96	AGCGCGATGATAAATTGTGTCGTGACGAGA
97	AACGCAAAGATAGCCGAACAAACCCTGAAC
98	GCCTCCCTCAGAATGGAAAGCGCAGTAACAGT
99	AAAGCACTAAATCGGAACCCTAATCCAGTT
100	GCCAGTTAGAGGGTAATTGAGCGCTTTAAGAA
101	AAGGCCGCTGATACCGATAGTTGCGACGTTAG
102	TTTTATTTAAGCAAATCAGATATTTTTTGT
103	CTTTTGCAGATAAAAACCAAAATAAAGACTCC
104	CCTAAATCAAAATCATAGGTCTAAACAGTA
105	AGACGACAAAGAAGTTTTGCCATAATTCGAGCTTCAA
106	AGAAAACAAAGAAGATGATGAAACAGGCTGCG
107	CGCGCAGATTACCTTTTTTAATGGGAGAGACT
108	CACAACAGGTGCCTAATGAGTGCCCAGCAG
109	GCGGAACATCTGAATAATGGAAGGTACAAAAT
110	TAAAAGGGACATTCTGGCCAACAAAGCATC
111	AATTGAGAATTCTGTCCAGACGACTAAACCAA
112	GCGAAAAATCCCTTATAAATCAAGCCGGCG
113	AACACCAAATTTCAACTTTAATCGTTTACC
114	TAAATCAAAATAATTCGCGTCTCGGAAACC
115	GAAACGATAGAAGGCTTATCCGGTCTCATCGAGAACAAGC
116	GCGAACCTCCAAGAACGGGTATGACAATAA
117	TTAGGATTGGCTGAGACTCCTCAATAACCGAT
118	ATCGCAAGTATGTAAATGCTGATGATAGGAAC
119	GCGGATAACCTATTATTCTGAAACAGACGATT
120	AAGGAAACATAAAGGTGGCAACATTATCACCG

121	ACCCTTCTGACCTGAAAGCGTAAGACGCTGAG
122	ATATTTTGGCTTTCATCAACATTATCCAGCCA
123	TCAAGTTTCATTAAAGGTGAATATAAAAGA
124	TCTAAAGTTTTGTCGTCTTTCCAGCCGACAA
125	TTCCAGTCGTAATCATGGTCATAAAAGGGG
126	AATACTGCCCAAAAGGAATTACGTGGCTCA
127	TTTATCAGGACAGCATCGGAACGACACCAACCTAAAACGA
128	TTGACAGGCCACCACCAGAGCCGCGATTTGTA
129	CTGTGTGATTGCGTTGCGCTCACTAGAGTTGC
130	GCGAGTAAAAATATTTAAATTGTTACAAAG
131	TAGAGAGTTATTTTCATTTGGGGATAGTAGTAGCATTA
132	CGAAAGACTTTGATAAGAGGTCATATTTCGCA
133	TCATCGCCAACAAAGTACAACGGACGCCAGCA
134	TTAACACCAGCACTAACAACTAATCGTTATTA
135	TTATTACGAAGAACTGGCATGATTGCGAGAGG
136	ACAACATGCCAACGCTCAACAGTCTTCTGA
137	CATTTGAAGGCGAATTATTCATTTTTGTTTGG
138	TGAAAGGAGCAAATGAAAAATCTAGAGATAGA
139	TGGAACAACCGCCTGGCCCTGAGGCCCGCT
140	TACCGAGCTCGAATTCGGGAAACCTGTCGTGCAGCTGATT
141	GTTTATTTTGTCACAATCTTACCGAAGCCCTTTAATATCA
142	ACAAACGGAAAAGCCCCAAAAACACTGGAGCA
143	GTTTATCAATATGCGTTATACAAACCGACCGTGTGATAAA
144	ACGGCTACAAAAGGAGCCTTTAATGTGAGAAT
145	GACCAACTAATGCCACTACGAAGGGGGTAGCA
146	CTCCAACGCAGTGAGACGGGCAACCAGCTGCA
147	ACCGATTGTCGGCATTTTCGGTCATAATCA
148	CAGAAGATTAGATAATACATTTGTCGACAA
149	TGCATCTTTCCCAGTCACGACGGCCTGCAG
150	TTAGTATCACAATAGATAAGTCCACGAGCA
151	GTTTTAACTTAGTACCGCCACCCAGAGCCA
152	TTAATGAACTAGAGGATCCCCGGGGGGTAACG
153	CTTTTACAAAATCGTCGCTATTAGCGATAG
154	ATCCCCCTATACCACATTCAACTAGAAAAATC
155	AGAAAGGAACAACTAAAGGAATTCAAAAAAA
156	AGCCACCACTGTAGCGCGTTTTCAAGGGAGGGAAGGTAAA
157	AACAAGAGGGATAAAAATTTTTAGCATAAAGC
158	GCCGTCAAAAAACAGAGGTGAGGCCTATTAGT
159	TGTAGAAATCAAGATTAGTTGCTCTTACCA
160	GAGAGATAGAGCGTCTTTCCAGAGGTTTTGAA
161	CCACCCTCATTTTCAGGGATAGCAACCGTACT
162	CTTTAATGCGCGAACTGATAGCCCCACCAG

163	CCAGGGTTGCCAGTTTGAGGGGACCCGTGGGA
164	CAAATCAAGTTTTTTGGGGTCGAAACGTGGA
165	ACGCTAACACCCACAAGAATTGAAAATAGC
166	TACGTTAAAGTAATCTTGACAAGAACCGAACT
167	TAATCAGCGGATTGACCGTAATCGTAACCG
168	TTTTCACTCAAAGGGCGAAAAACCATCACC
169	GCCTTAAACCAATCAATAATCGGCACGCGCCT
170	AATAGCTATCAATAGAAAATTCAACATTCA
171	CATCAAGTAAAACGAACTAACGAGTTGAGA
172	CAGGAGGTGGGGTCAGTGCCTTGAGTCTCTGAATTTACCG
173	AAATCACCTTCCAGTAAGCGTCAGTAATAA
174	CTCGTATTAGAAATTGCGTAGATACAGTAC
175	TTTACCCCAACATGTTTTAAATTTCCATAT
176	GTCGACTTCGGCCAACGCGCGGGGTTTTTC
177	CGTAAAACAGAAATAAAAATCCTTTGCCCGAAAGATTAGA
178	AGGCTCCAGAGGCTTTGAGGACACGGGTAA
179	GAGAAGAGATAACCTTGCTTCTGTTCGGGAGAAACAATAA
180	TTTAGGACAAATGCTTTAAACAATCAGGTC
181	AATACGTTTGAAAGAGGACAGACTGACCTT
182	CTTAGATTTAAGGCGTTAAATAAAGCCTGT
183	TAAATCGGGATTCCCAATTCTGCGATATAATG
184	AACAGTTTTGTACCAAAAACATTTTATTTC
185	CTGTAGCTTGACTATTATAGTCAGTTCATTGA
186	AACGCAAAATCGATGAACGGTACCGGTTGA

Supplementary Table 5 | Modifications to the rectangle

**Modified staple positions on the reference structure**
DNA PAINT docking strand on 3’: 5, 21, 23, 26, 50, 62, 76, 87, 110, 119, 121, 138, 157, 175, 180, 183, 184, 185
Biotin on 5’: 29, 61, 115, 131, 156, 179
**Modified staple positions on the sample structure**
Docking-strands for Gold-NP on 3’: 180, 183, 184<br>DNA PAINT docking strand on 3’: 4, 9, 25, 36, 54, 66
Biotin on 5’: 29, 61, 115, 131, 156, 179
**Modified staple positions on the *z* = 0 control structure**
DNA PAINT docking strands on 3’: 4, 9, 25, 36, 54, 66
Docking strands for permanent binding of Atto532-Oligos on 3’: 154, 157, 166, 171, 175, 180, 183, 184, 185, 186
Biotin on 5’: 29, 61, 115, 131, 156, 179
**Sequences of modified oligonucleotides and DNA-elongations on the rectangle**
Docking-strand for Gold-NP on 3’: AAAAAAAAAAAAAAAAAAAAAAAAA
Docking strands for permanent binding of Atto532-Oligos on 3’: TTTTCCTCTACCACCTACATCAC
Atto532-Oligo: Atto532-TTTGTGATGTAGGTGGTAGAGGAA
DNA PAINT docking strand on 3’: TTAAATGCCCG
9nt-Sequence of the imager strand: CGGGCATTT-Atto655

Supplementary Note 1:

In order to estimate the standard error (SE) of the height difference Δz plotted in Figure 3d, hereafter $\mathrm{SE}_{\Delta z}$, we considered three contributions $\mathrm{SE}_{z_{\mathrm{reference}}}$, $\mathrm{SE}_{z_{\mathrm{sample}}}$ and $\mathrm{SE}_{\mathrm{calibration}}$ representing the reference distribution's mean SE, the sample distribution's mean SE and the refractive index mismatch calibration SE illustrated in Supplementary Figure 4 respectively. In all three cases the SEs are multiplied by the confidence factor k.

Calculation of $SE_{\Delta z}$ by Gaussian error propagation yields:

$$\mathrm{SE}_{\Delta z} = \sqrt{\left(\frac{\partial \Delta z}{\partial z_{\mathrm{reference}}}\right)^2 \left(\mathrm{k} \times \mathrm{SE}_{z_{\mathrm{reference}}}\right)^2 + \left(\frac{\partial \Delta z}{\partial z_{\mathrm{sample}}}\right)^2 \left(\mathrm{k} \times \mathrm{SE}_{z_{\mathrm{sample}}}\right)^2 + 2\left(\frac{\partial \Delta z}{\partial \sigma_{\mathrm{calibration}}^{\mathrm{SE}}}\right)^2 (\mathrm{k} \times \mathrm{SE}_{\mathrm{calibration}})^2} \tag{1}$$

$$= \sqrt{\left(\mathrm{k} \times \mathrm{SE}_{z_{\mathrm{reference}}}\right)^2 + \left(\mathrm{k} \times \mathrm{SE}_{z_{\mathrm{sample}}}\right)^2 + 2(\mathrm{k} \times \mathrm{SE}_{\mathrm{calibration}})^2} \tag{2}$$

In equations (1) and (2) the influence of $\mathrm{SE}_{\mathrm{calibration}}$ is considered twice since it is acting on the reference measurement and the sample measurement independently.

To get the final value for the error bars the propagated error $\mathrm{SE}_{\Delta z}$ has again been multiplied by the confidence factor *k*. For our plots a value of *k*=3 has been chosen, corresponding to a confidence of ~99.7%.

Supplementary References

1. Schmied, J. J. *et al.* DNA origami nanopillars as standards for three-dimensional superresolution microscopy. *Nano Lett.* **13,** 781–785 (2013).

2. Su, L. *et al.* Visualization of molecular fluorescence point spread functions via remote excitation switching fluorescence microscopy. *Nat. Commun.* **6,** 6287 (2015).

3. Holzmeister, P. *et al.* Quantum yield and excitation rate of single molecules close to metallic nanostructures. *Nat. Commun.* **5,** 5356 (2014).

4. Egner, A. & Hell, S. W. in *Handbook Of Biological Confocal Microscopy* 404–413 (Springer US, 2006).

5. Huang, B., Jones, S. A., Brandenburg, B. & Zhuang, X. Whole-cell 3D STORM reveals interactions between cellular structures with nanometer-scale resolution. *Nat. Methods* **5,** 1047–1052 (2008).

6. Iinuma, R. *et al.* Polyhedra Self-Assembled from DNA Tripods and Characterized with 3D DNA-PAINT. *Science* **344,** 65–9 (2014).

7. Acuna, G. P. *et al.* Distance dependence of single-fluorophore quenching by gold nanoparticles studied on DNA origami. *ACS Nano* **6,** 3189–95 (2012).

8. Acuna, G. P. *et al.* Fluorescence enhancement at docking sites of DNA-directed self-assembled nanoantennas. *Science* **338,** 506–510 (2012).
