# Supplementary figures and images for: Shifting molecular localization by plasmonic coupling in a single-molecule mirage
Source: Nat Commun. 2017 Jan 11;8:13966. doi: 10.1038/ncomms13966 (PMC5512867; doi:10.1038/ncomms13966)

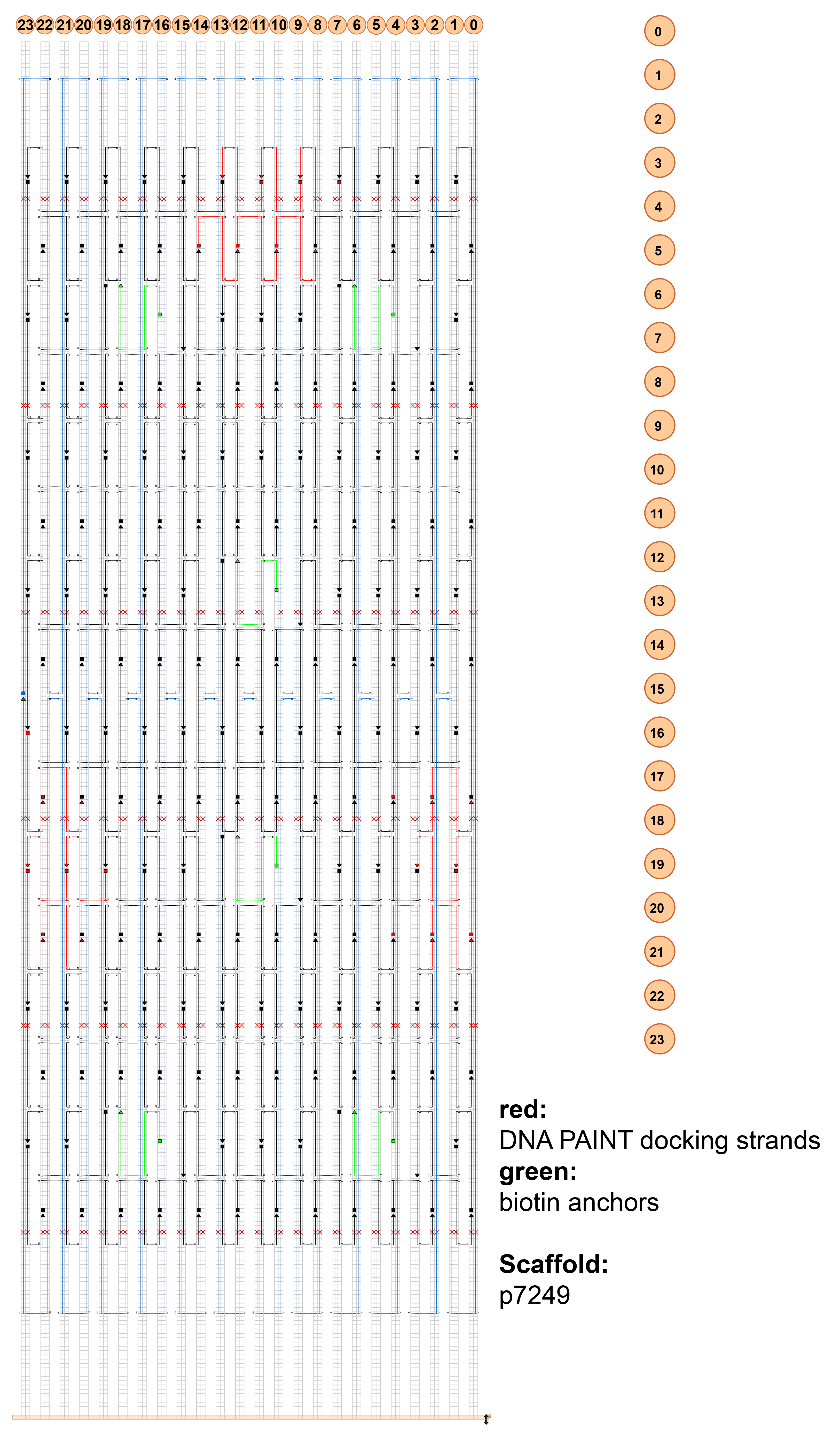

Supplement: Supplementary Data 2 — CaDNAno-design of the rectangular DNA origami for the reference structure. This is the detailed staple design of the DNA origami used to measure the height of the DNA origami surface. [file ncomms13966-s3.zip › Supplementary Data File 2.png]

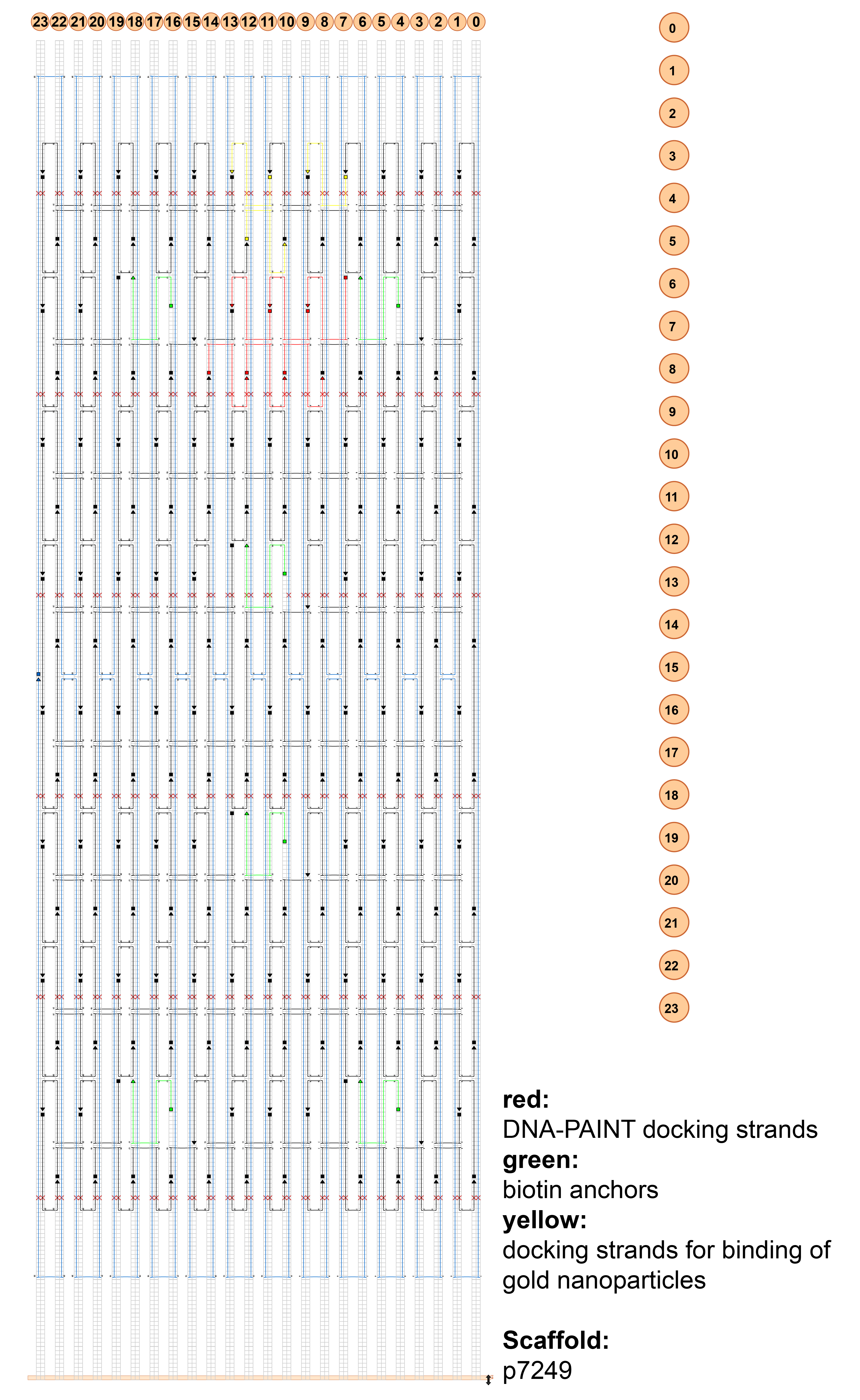

Supplement: Supplementary Data 3 — CaDNAno design of the rectangular DNA origami for the sample structure. This is the detailed staple design of the DNA origami used to measure shifted fluorescence signal compared to the DNA origami surface due to plasmonic coupling. [file ncomms13966-s4.zip › Supplementary Data File 3.png]

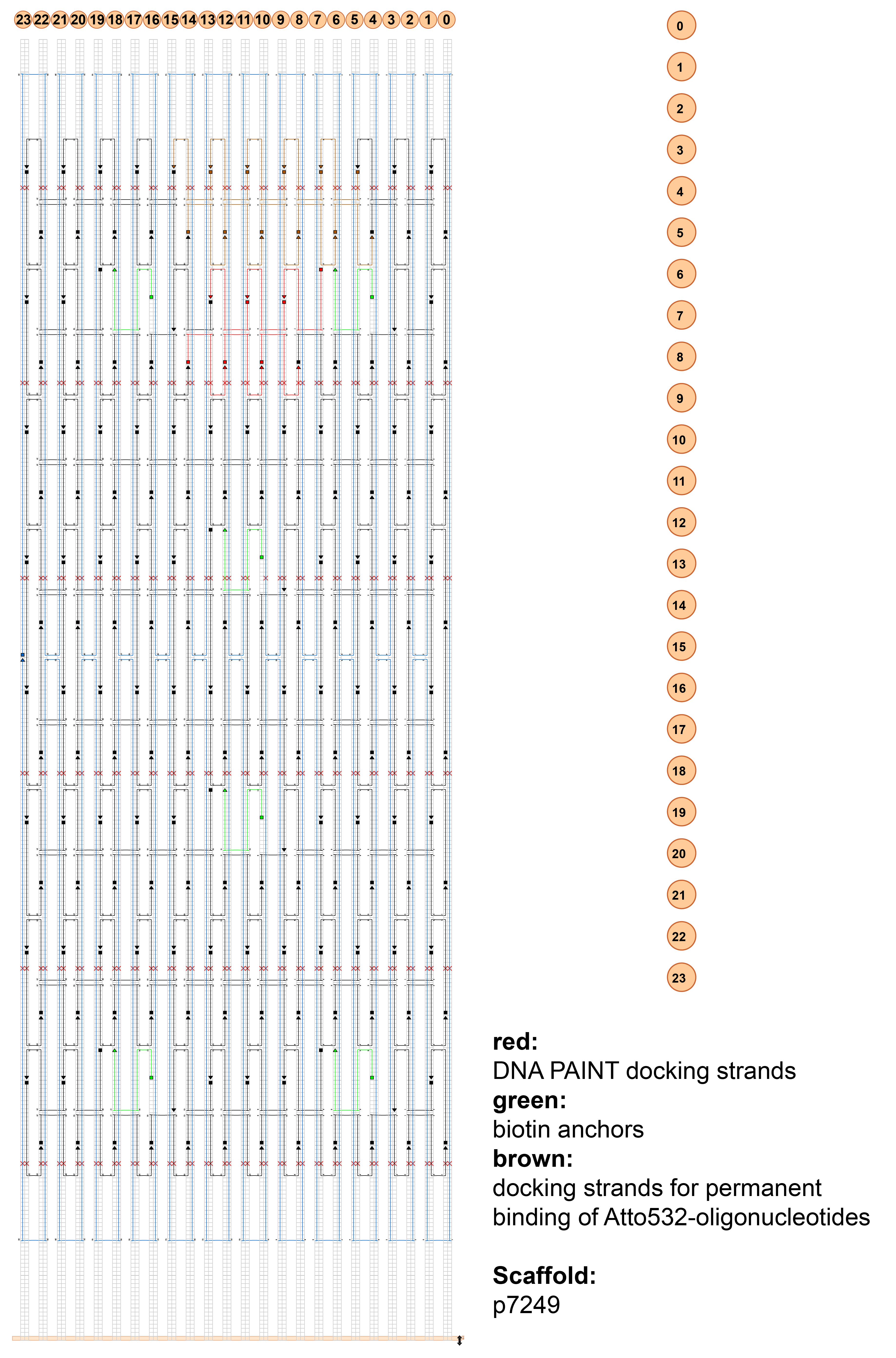

Supplement: Supplementary Data 4 — CaDNAno design of the rectangular DNA origami used for the control of the z = 0 baseline (Atto532-dyes instead of a gold nanoparticle (NP)). This is detailed staple design of the DNA origami representing "0 nm NP diameter". Because it has no NP that can be labeled with green dyes we attached these dyes directly on the DNA origami here to enable colocalization. [file ncomms13966-s5.zip › Supplementary Data File 4.png]
